# Study on the evaluation and influencing factors of contracted residents on the coordination of primary medical institutions

**DOI:** 10.3389/fpubh.2024.1307765

**Published:** 2024-06-04

**Authors:** Lingfeng Xu, Na Xu, Xiaoli Jiang, Haibo Peng, Yixuan Wu, Zihan Lang, Lifang Zhou, Dongping Ma, Zhongming Chen, Chengliang Yin, Qianqian Yu

**Affiliations:** ^1^School of Management, Shandong Second Medical University, Shandong, China; ^2^School of Public Health, Shandong Second Medical University, Shandong, China; ^3^School of Basic Medical Sciences, Shandong Second Medical University, Shandong, China

**Keywords:** family doctor contract service, China, primary medical institutions, coordination, primary care assessment tool

## Abstract

**Background:**

The implementation of family doctor contract service is a pivotal measure to enhance primary medical services and execute the hierarchical diagnosis and treatment system. Achieving service coordination among various institutions is both a fundamental objective and a central element of contract services.

**Objective:**

The study aims to assess residents’ evaluations and determining factors related to the coordination of health services within primary medical institutions across different regions of Shandong Province. The findings intend to serve as a reference for enhancing the coordination services offered by these institutions.

**Methods:**

The study employed a multi-stage stratified random sampling method to select three prefecture-level cities in Shandong Province with different economic levels. Within each city, three counties (districts) were randomly sampled using the same method. Within each county (district), three community health service centers and township health centers implementing family doctor contract services were selected randomly. Face-to-face questionnaire surveys were conducted with contracted residents using the coordination dimension of the revised Primary Care Assessment Tools Scale (PCAT) developed by the research team. Data analysis was conducted using such methods as one-way analysis of variance and multiple linear regression.

**Results:**

The sample included 3,859 contracted residents. The coordination dimension score of primary medical institutions averaged 3.41 ± 0.18, with the referral service sub-dimension scoring 3.60 ± 0.58 and the information system sub-dimension scoring 3.34 ± 0.65. The overall score of the referral service sub-dimension surpassed that of the information system sub-dimension. Regression results indicated that the city’s economic status, the type of contracted institutions, gender, education, marital status, income, occupation, health status, and endowment insurance payment status significantly influenced the coordinated service score of primary medical institutions (*p* < 0.05).

**Conclusion:**

The coordination of primary medical institutions in Shandong Province warrants further optimization. Continued efforts should focus on refining the referral system, expediting information infrastructure development, enhancing the service standards of primary medical institutions, and fostering resident trust. These measures aim to advance the implementation of the hierarchical diagnosis and treatment and two-way referral system.

## 1 Introduction

According to the data from the 2022 China Health Statistical Yearbook, the number of primary medical and health institutions in China rose from over 920,000 in 2015 to more than 970,000 in 2021. During the same period, the total consultations decreased from 4.34 billion in 2015 to 4.25 billion in 2021. The number of hospitals increased from 27,500 in 2015 to 36,500, with total consultations rising from 3.08 billion to 3.88 billion (1). Hospitals have increasingly taken on roles that were originally intended for basic medical and health institutions, leading to a widening gap that is not conducive to the efficient allocation of healthcare resources. This situation also makes healthcare access more expensive and inadequate to address the challenges brought by China’s aging population (2, 3). In 2016, the “Deepening China’s Medical and Health System Reform-Building a Value-Based Quality Service Delivery System” jointly conducted by the World Bank, the World Health Organization, and other parties, highlighted significant achievements in China’s medical reform. However, it also emphasized the need for a series of measures to control escalating healthcare costs. These measures include strengthening primary healthcare services, implementing a hierarchical diagnosis and treatment system, and enhancing healthcare quality. The report centralizes the recommendation to encourage greater utilization of primary healthcare services and reduce reliance on costly hospital services. It proposed a People-Centered Integrated Care (PCIC) approach, which focuses on the health needs of residents and their families through a robust primary healthcare system. This approach is designed to deliver comprehensive and personalized healthcare services, encompassing health education, prevention, therapeutic intervention, rehabilitation, and palliative care (4). These services were integrated to meet the diverse health requirements of different resident groups and facilitate coordination among healthcare institutions at all levels to offer a seamless, lifelong continuum of care (5).

In many countries, primary health care is usually provided by general practitioners, also known as family doctors, and has been shown to save costs and improve the health of the population (6–8). On June 6, 2016, the State Council of China issued the “Guiding Opinions on Promoting Family Doctor Contract Service,” marking the commencement of the nationwide exploration of the family doctor contract service model (9). The implementation of family contract services is a crucial step in enhancing the quality of primary medical services and implementing the hierarchical diagnosis and treatment system. It represents significant national efforts to enhance the basic medical service system. In China, the primary practice of family doctor contract services involves policy promotion, encouraging residents to voluntarily enter into service agreements with family doctors, guiding certain basic medical service needs to the grassroots level, and ensuring a rational distribution of medical needs among institutions at varying levels (10). This approach aims to alleviate issues like “difficult and expensive medical treatment.” Given China’s substantial older adult population, its rapidly aging demographic, and the high prevalence of chronic diseases, disability, and dementia among the older adult, there is a significant demand for basic medical services. The family doctor contract service system can offer suitable prevention, medical treatment, healthcare, rehabilitation, hospice, and other services for the older adult, enhancing their health status and quality of life while effectively addressing the medical resource scarcity resulting from aging (11).

Coordination is a central principle of primary healthcare, particularly in general practice, and is a key feature in achieving the economic effectiveness of basic medical services. Coordinated care services are a collaborative and integrated process of health services tailored to the individual needs of each patient, providing coordinated services in prevention, medical treatment, rehabilitation, and health promotion. It adheres to a people-centered service philosophy, focusing on the physical and psychological health needs of patients, ensuring that they find the most appropriate health service providers for specific health issues (12, 13). Coordination within primary medical institutions is a crucial element of care coordination, ensuring that basic medical services are part of an open and cooperative system with organic connections to higher-level medical institutions, specialists, and other health organizations. This coordination aims to establish a robust collaborative system among multiple health service providers, which includes a clear division of responsibilities among medical institutions, smooth referral processes, and shared medical information. It seeks to minimize unnecessary tests and treatments, enhance the effectiveness of visits, allocate health care resources rationally, and improve the cost-effectiveness of general medical care services (14–16).

After several years of practice, family doctor contract services have positively contributed to optimizing the utilization of primary medical resources, easing the challenges associated with seeking care in large hospitals, lowering healthcare expenses, and enhancing residents’ health status (17, 18). Nonetheless, China currently grapples with issues such as an underdeveloped hierarchical diagnosis and treatment system, an immature two-way referral system, non-standardized family doctor practice qualification certification standards, subpar service quality, a disproportionate trend of patient transfers upwards rather than downwards, non-smooth referral channels, and unsatisfactory grassroots medical experiences (19, 20). In the context of domestic and foreign research on primary medical and health services, foreign studies predominantly emphasize coordination, humanization, and accessibility, whereas domestic studies center on comprehensiveness, accessibility, and humanization, often neglecting the research on service coordination (21, 22). Based on the above, this study employs the coordination dimension of the Primary Care Assessment Tool (PCAT) scale, which includes the two sub-dimensions of referral service and information system, to evaluate the coordination of services provided by primary health care institutions. The PCAT, developed by Dr. Barbara Starfield and Dr.Leiyu Shi in Johns Hopkins University, is a widely utilized instrument that measures several key dimensions of primary care delivery, including first-contact, continuity of care, comprehensiveness of care, and coordination of care, and is designed to assess the quality of primary health care services. It has been adapted for use in multiple countries, translated into various languages, and localized to align with different cultural and health system contexts. The reliability and validity of the questionnaire have been substantiated across diverse research and application settings (23–26). Utilizing the PCAT for this survey not only aims to address the deficiency in research on the coordination of health care services in China but also holds significant practical value. It contributes to the refinement of China’s tiered diagnosis and treatment system, enhances the quality of services at primary health care institutions, meets the health service demands of patients, and improves patient satisfaction and the overall health status of the population.

## 2 Subjects and methods

### 2.1 Subjects of survey

In January 2021, the research team conducted a field survey in Shandong Province. Employing a multi-stage stratified random extraction approach, the research categorized the economic development levels of 16 cities in Shandong Province into three tiers: high, medium, and low. A city was chosen from each tier, and three counties (including cities and districts) were selected from each city using the same methodology. Within each selected county (city or district), three community health service centers and three township health centers were chosen to offer contracted family doctor services, resulting in a total of 27 community health service centers and 27 township health centers. Approximately 60 residents were chosen from each primary medical institution based on the registration records, and all these residents gave informed consent and participated voluntarily.

### 2.2 Research method

#### 2.2.1 Research tool

This study employs the revised Chinese version of the Primary Care Assessment Tool (PCAT), which evaluates patient acceptance of specific services throughout the provision of medical and health services, thereby offering an objective assessment of the quality of contracted family doctor services in China. The coordination dimension comprises two sub-dimensions: the referral service sub-dimension, which measures the level of cooperation between the primary medical institution and higher-level medical institutions, and the information system sub-dimension, which assesses the ease of accessing patient medical record information. The scale utilizes a 4-level Likert scoring method for residents under contract to respond and rate. “Definitely will” receives 4 points, “probably will” earns 3 points, “probably will not” is assigned 2 points, “Definitely will not” is granted 1 point, and “not sure/do not know” is scored with 2 points. The two items mentioned above, “Have you sought medical treatment at a large hospital or specialized hospital” and “Is referral by a contracted institution required before visiting a large hospital or specialized hospital,” do not employ the Likert scale format. Instead, they offer response options of “yes,” “no,” and “uncertain/do not know,” and are not factored into the scale scoring. The coordination score is calculated as the average of all item scores across the two dimensions. A higher score indicates better coordination services provided by the institution.

#### 2.2.2 Reliability and validity testing

The questionnaire was tested for reliability using Cronbach’s alpha coefficient, and structural validity was tested by applying exploratory factor analysis. The results of the reliability test showed that the Cronbach’s alpha coefficient of the scale Coordination Dimension-Referral Services was 0.905 (>0.700), and the Cronbach’s alpha coefficient of the scale Coordination Dimension-Information System was 0.790 (>0.700), which indicated a good reliability. The results of exploratory factor analysis showed that the KMO test statistic for the scale coordination dimension-referral service dimension was 0.926, the Bartlett’s globule test statistic was 12832.252 (*p* < 0.001), the KMO test statistic for the scale coordination dimension-information system dimension was 0.781, and Bartlett’s spherical test statistic of 5394.140 (*p* < 0.001), suitable for factor analysis. All of these met the fit criteria, indicating that the questionnaire has good validity. The questionnaire also demonstrated good reliability and validity in previous related studies by the subject group (27).

#### 2.2.3 Statistical method

The questionnaire was uniformly coded, and the database was established by SPSS26.0, and the data were entered and quantitatively analyzed by descriptive analysis. Normally distributed measurement data were expressed as M ± SD (mean ± standard deviation). The t-test or one-way analysis of variance was used to analyze the differences in the evaluation of the coordination service of primary medical institutions among different contracted residents. The influencing factors of the evaluation of the coordination service of primary medical institutions were analyzed by multiple linear regression analysis. The significance level for the tests was set at *p* < 0.05, indicating statistical significance.

## 3 Results

### 3.1 Characteristic of subjects

The survey distributed a total of 4,000 questionnaires, with 3,859 valid questionnaires returned, resulting in an effective response rate of 96.48%. Among these, 1,773 respondents (45.9%) were residents affiliated with community health service centers, while 2,086 respondents (54.1%) were residents affiliated with township health centers. The survey population consisted of slightly more females than males, with 2,057 females (53.3%). The age group of 60 years and above was the largest, comprising 1,244 individuals (32.2%). The majority of respondents had attained education levels of primary school or below and junior high school, accounting for 1,204 individuals (31.2%) and 1,276 individuals (33.1%) respectively. The married population accounted for 3,257 individuals (84.4%), while 3,393 respondents (87.9%) were employed. Approximately 45.7% of respondents had a monthly income of less than 2,000 yuan. Regarding self-rated health status, 2,659 individuals (68.9%) described their health as “very good” or “good.” The number of individuals with chronic diseases was 1,370 (35.5%). On average, the number of visits to the contracted healthcare institution in the past month was 1.53 ± 2.14 times.

### 3.2 Evaluation of the coordination score of primary medical service institutions

Contracted residents’ overall score for the coordination service of primary medical institutions was 3.41 ± 0.57 points. Specifically, the evaluation score for the referral service dimension was 3.60 ± 0.58 points, while the evaluation score for the information system dimension was 3.34 ± 0.65 points. The average score for the referral service exceeded that of the information system. Within the referral service dimension, the highest score, 3.75 ± 0.55, was recorded for “Do you get the results of your lab tests?” while the lowest score, 3.35 ± 0.85, pertained to “Whether there are specialists from higher level hospitals in your community?” Within the information system dimension, the highest score, 3.57 ± 0.64, was achieved for “Did your family doctor refer to your previous medical records at each visit?” while the lowest score, 2.92 ± 1.03, related to “Can you see your medical records on the mobile client?”, as shown in Table 1.

**Table 1:** Scores of contracted residents on coordinated services of primary medical institutions (M ± SD).

Dimensions	Content description	Score
Coordination	Sum of two sub dimensions of referral service and information system	3.41 ± 0.57
Coordination-referral	Do you get the results of your lab tests?	3.75 ± 0.55
	Did your family doctor suggest you go to the specialist or special service?	3.50 ± 0.65
	Did your family doctor know you made these visits to the specialist or special service?	3.53 ± 0.73
	Did your family doctor discuss with you different places you could have gone to get help with that problem?	3.45 ± 0.74
	Did your family doctor help you make the appointment for that visit?	3.46 ± 0.77
	Did your family doctor write down any information for the specialist about the reason for the visit?	3.54 ± 0.73
	After you went to the specialist or special service, did your family doctor talk with you about what happened at the visit?	3.55 ± 0.73
	Did your family doctor seem interested in the quality of care you received from that specialist or specialist service?	3.44 ± 0.80
	When the disease is treated and the condition is stable, whether it will be returned to community institutions for rehabilitation?	3.55 ± 0.67
	Whether there are specialists from higher level hospitals in your community?	3.35 ± 0.85
	Average sub-dimension scores	3.60 ± 0.58
Coordination-information systems	When you go to your family doctor, do you bring any of your own medical records, such as shot records or reports of medical care you had in the past?	3.43 ± 0.85
	When you go to your contracted institution, is your medical record always available?	3.43 ± 0.74
	Did your family doctor refer to your previous medical records at each visit?	3.57 ± 0.64
	Can you see your medical records on the mobile client?	2.92 ± 1.03
	Average sub-dimension scores	3.34 ± 0.65

### 3.3 Difference analysis of PCAT coordination service evaluation scores of contracted residents with different characteristics

The study identified several factors that were statistically significant in relation to the PCAT coordination dimension scores of contracted residents. These factors included the city’s economic level, contracting institutions, age, education level, marital status, occupation, income, self-rated health status, diagnosis of chronic diseases, medical insurance coverage, and endowment insurance availability. The PCAT coordination dimension scores for contracted residents exhibited statistical significance (*p* < 0.05), as shown in Table 2.

**Table 2:** Comparison of PCAT coordination scores of contracted residents with different characteristics (M ± SD, points).

Variable	Classification	*n*	PCAT score	*t*(*F*)	*p*-value
Local economy level	Good	1,333	3.56 ± 0.54	434.123^a^	<0.001*
	General	1,428	3.57 ± 0.46		
	Poor	1,098	3.02 ± 0.56		
Contracted institution	Township Health Center	2086	3.48 ± 0.51	8.345	<0.001*
	Community Health Center	1773	3.33 ± 0.62		
Gender	Male	1802	3.46 ± 0.56	4.720	<0.001*
	Female	2057	3.37 ± 0.58		
Age	Under18	39	3.30 ± 0.72	31.244^a^	<0.001*
	18 ~ 29	359	3.17 ± 0.74		
	30 ~ 39	507	3.56 ± 0.51		
	40 ~ 49	900	3.48 ± 0.52		
	50 ~ 59	810	3.47 ± 0.51		
	≥60	1,244	3.33 ± 0.58		
Education	Primary school and below	1,204	3.25 ± 0.58	52.923^a^	<0.001*
	Junior high school	1,276	3.53 ± 0.51		
	High school / technical secondary school	657	3.50 ± 0.51		
	Junior college	358	3.51 ± 0.57		
	Bachelor degree or above	364	3.25 ± 0.70		
Marital status	Unmarried	346	3.10 ± 0.74	49.560^a^	<0.001*
	Married	3,257	3.46 ± 0.54		
	Divorce	36	3.24 ± 0.62		
	Widowed	220	3.24 ± 0.58		
Occupation	In service personnel	3,393	3.44 ± 0.55	66.463^a^	<0.001*
	School Students	214	2.98 ± 0.75		
	Retired personnel	252	3.44 ± 0.57		
Personal monthly income (RMB yuan)	<2000	1765	3.31 ± 0.61	52.381^a^	<0.001*
	2000 ~ 4,999	1,549	3.51 ± 0.51		
	≥5,000	545	3.45 ± 0.56		
Self-rated health status	Very good	1,028	3.55 ± 0.53	32.947^a^	<0.001*
	Good	1,631	3.39 ± 0.56		
	General	957	3.34 ± 0.59		
	Poor	228	3.17 ± 0.58		
	Very poor	15	3.06 ± 0.61		
Is chronic disease diagnosed	Yes	1,370	3.37 ± 0.57	−3.562	<0.001*
	No	2,489	3.44 ± 0.57		
Health insurance	Yes	3785	3.42 ± 0.57	−3.243	0.002*
	No	74	3.15 ± 0.72		
Endowment insurance	Yes	3,272	3.44 ± 0.55	−7.236	<0.001*
	No	586	3.24 ± 0.65		

a indicates *F*-value, ^*^indicates *p* < 0.05.

### 3.4 Multiple linear regression analysis of the impact of coordinated service score of primary medical institutions

The variables showing statistical significance in the single-factor analysis were used as independent variables, while the coordination service score was set as the dependent variable in a multiple linear regression analysis. Dummy variables were set for economic level of the city, education level, and marital status, while income and age were entered as original values. The results indicated that the economic development of the city, contracted healthcare institution, gender, education, marital status, income, occupation, health status, and whether one pays social security contributions were influencing factors on the coordination service score of grassroots medical institutions. Compared to cities with better economic conditions, cities with lower economic development had lower coordination service scores. Community health service centers had lower coordination scores compared to township health centers. Individuals with higher income had lower coordination service scores compared to those with lower income. The coordination service scores were higher for employed individuals compared to students. Individuals with “very good” health status had the highest coordination scores. Those with endowment insurance scored higher compared to those without endowment insurance. Refer to Table 3 for details.

**Table 3:** Multiple linear regression analysis of the influence of coordination service score.

Variable	*β*	*SE*	*Sβ*	*t-value*	*P-value*	95%CL
Local economy level (taking the good as a reference)						
General=1	-0.003	0.019	-0.003	-0.173	0.863	-0.041~0.035
Poor=2	-0.479	0.022	-0.378	-21.685	<0.001*	-0.522~-0.436
Contracted institution (taking the community health center as a reference)						
Township health Center=1	0.086	0.018	0.075	4.875	<0.001*	0.051~0.12
Gender (taking the female a reference)						
Male=1	0.040	0.017	0.035	2.378	0.017*	0.007~0.072
Age(entry at original value)	-0.001	0.001	-0.019	-0.787	0.431	-0.002~0.001
Education (taking the primary school and below as a reference)						
Junior high school=1	0.076	0.024	0.062	3.199	0.001*	0.029~0.122
High school / technical secondary school=2	0.025	0.029	0.017	0.858	0.391	-0.032~0.083
Junior college=3	0.108	0.037	0.055	2.933	0.003*	0.036~0.181
Bachelor degree or above=4	0.017	0.041	0.009	0.415	0.679	-0.063~0.097
Marital status (taking the unmarried as a reference)						
Married=1	0.166	0.046	0.105	3.585	<0.001*	0.075~0.257
Divorce=2	-0.065	0.094	-0.011	-0.686	0.493	-0.250~0.120
Widowed=3	0.166	0.060	0.068	2.778	0.006*	0.049~0.284
Personal monthly income (Entry at original value)	-8×10^-6^	0.000	-0.059	-3.913	<0.001*	0.000~0.000
Occupation (taking the service personnel as a reference)						
School students	-0.183	0.058	-0.073	-3.167	0.002*	-0.296~-0.070
Retired personnel	0.000	0.035	0.000	0.012	0.991	-0.069~0.069
Self-rated health status (taking the very good as a reference)						
Good	-0.167	0.021	-0.144	-8.098	<0.001*	-0.208~-0.127
General	-0.196	0.025	-0.148	-7.858	<0.001*	-0.245~-0.147
Poor	-0.250	0.040	-0.103	-6.196	<0.001*	-0.329~-0.171
Very poor	-0.418	0.131	-0.045	-3.192	0.001*	-0.674~-0.161
Is chronic disease diagnosed (taking the no as a reference)						
Yes	0.039	0.020	0.033	1.922	0.055	-0.001~0.079
Health insurance (taking the yes as a reference)						
No	-0.068	0.061	-0.016	-1.125	0.261	-0.187~0.051
Endowment insurance (taking the yes as a reference)						
No	-0.051	0.026	-0.032	-1.992	0.046*	-0.101~-0.001
Constant	3.489	0.065	—	53.899	0.000	3.362~3.616

^*^indicates *p*<0.05.

## 4 Discussion

The study reveals that residents with different demographic characteristics have varied perceptions of the coordination of medical services, which may reflect disparities in the capacity of medical institutions and the needs of patients. Prefecture-level cities with better economies tend to have higher coordination service scores compared to those with weaker economies, likely due to more substantial financial allocations from higher authorities, ensuring stable funding for software, hardware, and personnel, which in turn enhances the overall competence of medical institutions and increases patient trust (28, 29). Residents affiliated with community health service centers rate the coordination dimension lower than those from township health centers, possibly because urban community residents have easier access to large medical facilities and prefer to seek care there when facing health issues (30), and also because urban residents are more sensitive to the quality of medical services and less sensitive to costs (31). Females score lower on the coordination dimension than males, possibly due to their better health status and fewer medical visits, leading to less utilization of medical services and thus lower coordination scores (32). Older adults who have paid into a pension insurance scheme have higher coordination scores, likely because they have greater financial capacity to meet their medical needs, reflecting the impact of medical demand on coordination scores.

Upon examining the two sub-dimensions and specific items of coordination, the referral service sub-dimension scores higher than the information system sub-dimension, indicating some progress in China’s bidirectional referral system. Following the explicit introduction of the tiered diagnosis and treatment concept in China in 2015, bidirectional referral was established as a component of this system, and a series of policies were progressively implemented to ensure the system’s effectiveness. These policies included initiatives such as primary care first diagnosis, urgent and non-urgent disease treatment, urban medical consortiums, county medical communities, specialty alliances, telemedicine collaborative networks, family doctor contract services, and internet medical services. These measures have led to improvements in the quality of primary care and the advancement of the tiered diagnosis and treatment system to a certain extent. However, there are still areas that require attention. In the sub-dimension of referral services, the item “Whether there are specialists from higher level hospitals in your community?” received the lowest score, indicating a scarcity of specialists providing consultations at primary health care institutions. This suggests that the support from higher-level hospitals to their subordinate institutions is insufficient. The low score reflects the need for enhanced support mechanisms to ensure that primary care institutions benefit from the expertise and resources of higher-level hospitals, thereby improving the overall coordination and quality of care within the tiered health system. The reasons may include a low proportion of individuals at higher-level hospitals who are aware of and participate in assistance efforts, and a lack of substantive incentives for those who do participate, affecting their motivation to provide services (33). Several items within the referral service sub-dimension, such as “Did your family doctor seem interested in the quality of care you received from that specialist or specialist service?” “Did your family doctor discuss with you different places you could have gone to get help with that problem?” and “Did your family doctor help you make the appointment for that visit?” scored low, indicating that patient-doctor communication is often limited to disease-related matters. It is essential to not only improve clinical service capabilities but also to transform the patient-doctor relationship, shifting the focus from disease-centered to health-centered care (34, 35).

In addition, the study found that healthcare informatization in China is still slow, especially in less developed areas and township health centers. On one hand, due to insufficient financial support from higher authorities, it is challenging for institutions to develop their infrastructure independently. On the other hand, the lack of information management specialists at the grassroots level, coupled with low proficiency in operating information systems and limited internet literacy, restricts the effective use of medical information systems (36). Additionally, a significant portion of the contracted residents are older adult and not familiar with the operation of smartphones. Even if available, smartphones cannot be widely adopted among this demographic group, thus reducing the score for the information system dimension.

The study has several limitations that should be acknowledged. Firstly, the selection of three prefecture-level cities in Shandong Province for this study only reflects the health service conditions within these cities and may not be extrapolated to other regions outside of Shandong. Future research should consider a broader selection of samples from areas with varying geographical locations and economic development levels to enhance the external validity of the findings. Secondly, the current evaluation of health service coordination is based on a cross-sectional survey, which captures the state of coordination at a single point in time and does not reveal the impact of health policies on coordination. Future studies could benefit from interventional research or time series analyses to assess changes in health service coordination following the implementation of specific health policies, thereby improving the comprehensiveness of the evaluation. Lastly, existing research has primarily focused on objective indicators, such as the utilization of medical services by residents or the supply behavior of healthcare providers, which do not measure the subjective perception of coordination by residents. Future research could aim to construct a multidimensional measurement index system for health service coordination that includes both subjective and objective aspects, enhancing the accuracy of the evaluation.

## 5 Policy implications

### 5.1 Improve the two-way referral channel and give play to the synergistic effect of different institutions

Firstly, health administrative departments should promote the establishment of medical alliances among hospitals at different levels and transform them into closely cooperating communities of shared interests. This entails unified management in six aspects: personnel, medical services, finance, material allocation, health services, and information resources within the medical alliances. It involves establishing a mechanism for division of labor and cooperation among various medical institutions, formulating principles and procedures for bidirectional referral of common diseases, and proposing technical solutions for the graded diagnosis and treatment of chronic diseases. Simultaneously, there should be increased financial investment in medical institutions, especially those at higher levels, to alleviate their economic pressure and address the interests between medical institutions at different levels (37). Secondly, it is essential to improve policies and systems related to tiered diagnosis and treatment, such as adjustments in medical service pricing, differentiation in medical insurance payments, salary distribution, and performance evaluation. Through institutional arrangements, various levels of medical institutions should implement their respective functional positioning. Regarding medical service pricing and medical insurance payments, attracting patients to seek treatment at grassroots levels can be achieved by reducing service prices at grassroots medical institutions and increasing the compensation ratio of medical insurance for grassroots institutions. Concerning salary distribution and performance evaluation, establishing appropriate performance assessment mechanisms and refining referral standards are crucial. This involves integrating the performance of medical service providers from higher-level hospitals with patients exhibiting characteristics suitable for downward referral. Providers who meet referral criteria but fail to refer patients downward should be assessed, and supervisory mechanisms such as deducting performance should be implemented to encourage eligible patients to be referred downward.

### 5.2 Improve the level of informatization and promote the development of grassroots medical institutions enabled by the internet

Firstly, there should be a primary focus on enhancing the level of informatization in primary healthcare institutions. Leveraging “Internet+” technology as its foundation, a unified health information platform should be established, with residents’ electronic records and electronic medical records serving as its core basis. Within this platform, patients should be able to conduct primary consultations at any medical institution within the medical consortium. They should also have the capability to schedule appointments for check-ups or referrals to higher-level hospitals, thereby fully accessing the high-quality services provided by tertiary hospitals. Secondly, the establishment of a medical information integration platform is essential. This platform should eliminate information barriers between different levels of healthcare institutions, facilitating doctors’ access to patient data and medical histories. It should achieve the seamless circulation of electronic medical records and health records across institutions of varying levels. Additionally, patients should have the convenience of accessing their own check-up reports or medical records through a mobile application (38). Thirdly, regular information technology training programs should be implemented for personnel working in primary healthcare service institutions. These programs should aim to enhance their information literacy and proficiency in system operations, while also encouraging the utilization of information systems to improve service quality and efficiency. Lastly, it is crucial to gather the requirements of older adult individuals and develop tailored modes, such as those featuring larger fonts, bigger icons, and simplified and easily understandable reports. This approach aims to enhance older adult residents’ utilization of information.

### 5.3 Improve the service capacity of primary medical institutions and enhance the trust of residents

Efforts to enhance the diagnosis and treatment capabilities of primary medical institutions are essential to meet the demand for basic healthcare services. The current bottleneck in implementing hierarchical diagnosis and two-way referral policies primarily stems from the limited-service capacity of these institutions (37). To elevate the quality of medical services at the grassroots level, the following measures can be taken: First, attracting college graduates to grassroots services: Encourage recent college graduates to serve at the grassroots level by offering improved compensation packages, enhanced welfare benefits, elevated social status, and other incentives. This strategy aims to expand the pool of talented healthcare professionals at the grassroots level. Second, reforming incentive mechanisms for superior hospital staff: Adjust incentive mechanisms to motivate healthcare personnel from superior hospitals to actively engage at the grassroots level. For instance, modify promotion policies for professional titles to favor those who provide assistance at the grassroots level. Introduce mandatory requirements for professional title promotion for resident staff stationed at grassroots institutions. Additionally, provide distinct and timely rewards for outstanding performance in terms of salary, fostering a sense of accomplishment and motivation among stationed staff (39). Third, strengthen drug support at grassroots level: Enhance the capacity of grassroots-level healthcare facilities to supply essential drugs. This involves a rational allocation of medications, especially for common diseases, frequently-occurring conditions, and chronic illnesses. Ensuring a consistent supply of medications improves drug accessibility for the general public and expands the range of drugs available at grassroots institutions (40).

## Data availability statement

The raw data supporting the conclusions of this article will be made available by the authors, without undue reservation.

## Ethics statement

The studies involving humans were approved by Medical Ethics Committee of Shandong Second Medical University. The studies were conducted in accordance with the local legislation and institutional requirements. The Ethics Committee/Institutional Review Board waived the requirement of written informed consent for participation from the participants or the participants’ legal guardians/next of kin because the subjects were not potentially at risk and the survey was completed with the assistance of the administrator of the local healthcare facility.

## Author contributions

LX: Writing – original draft, Writing – review & editing, Data curation, Investigation. NX: Writing – review & editing, Investigation, Data curation. XJ: Writing – review & editing, Investigation, Data curation. HP: Supervision, Writing – review & editing, Investigation. YW: Data curation, Writing – review & editing. ZL: Writing – review & editing, Investigation. LZ: Writing – review & editing. DM: Writing – review & editing. ZC: Writing – review & editing. CY: Writing – review & editing, Conceptualization. QY: Writing – review & editing, Supervision, Conceptualization, Funding acquisition.
