# Supplementary material for: Study on the evaluation and influencing factors of contracted residents on the coordination of primary medical institutions
Source: Front Public Health. 2024 Jun 4;12:1307765. doi: 10.3389/fpubh.2024.1307765 (PMC11183267; doi:10.3389/fpubh.2024.1307765)
Supplement: Supplementary file 1 [file Table_1.DOCX]

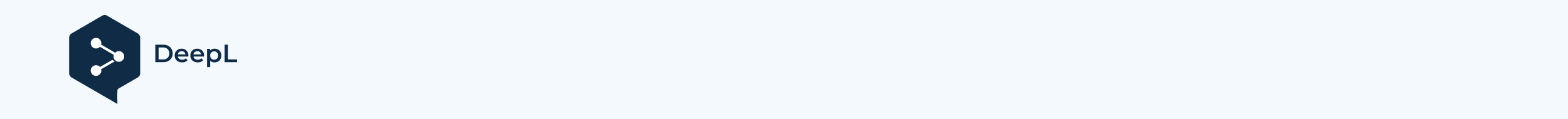


Subscribe to DeepL Pro to edit this document.
Visit [www.DeepL.com/pro](https://www.deepl.com/pro?cta=edit-document) for more information.

**Questionnaire for evaluating the effectiveness of contracted family doctor services**

Hello!

This questionnaire survey is to find out your experience of visiting family doctors' contracted services in order to better improve the quality of family doctor contracted services. Your participation is purely **voluntary**, the survey is anonymous, and all information you provide will be kept confidential and used for research purposes only. Your valuable opinions will have a direct contribution to the improvement of primary healthcare services.

**Questionnaire filling requirements: Please fill in the answers on the right hand side of the response area**

**I. Basic information on individual residents**

| **serial number** | **Questions and Options** | **responsive** |
| --- | --- | --- |
| 1 | Gender: ①Male ②Female |  |
| 2 | Age:____Years Old |  |
| 3 | Education level: ① Primary school and below ② Junior high school ③ High school / technical secondary school ④ Junior college ⑤ Bachelor degree and above |  |
| 4 | Marital Status: ①Unmarried ②Married ③Divorced ④Widowed |  |
| 5 | Occupation: ①Managers ②Professionals and technicians ③Officers ④Commercial/service workers ⑤Agricultural, forestry, fishery and water conservancy workers ⑥ Production and transportation equipment operators ⑦ School students ⑧ Retirees ⑨ Others |  |
| 6 | Your personal income: ____￥/month |  |
| 7 | How do you feel about your physical health:   1. Very good ② Good ③ General ④ Poor ⑤ Very poor |  |
| 8 | Do you have a diagnosed chronic disease? ① Yes ② No (skip to Q10) |  |
| 9 | Which of the following chronic diseases (multiple choice): ① hypertension ② diabetes mellitus ③ intervertebral disc disease ④ cerebrovascular disease ⑤ gastroenteritis ⑥ ischemic heart disease ⑦ rheumatoid arthritis ⑧ Chronic obstructive pulmonary disease ⑨ Others (please specify) __________ |  |
| 10 | Your enrollment in health insurance (check all that apply):  ①No medical insurance ②New rural cooperative medical insurance③Basic medical insurance for urban workers ④Medical insurance for urban and rural residents  ⑤ Urban residents' medical insurance ⑥ Commercial medical insurance ⑦ Others (please specify) __________ |  |
| 11 | Your participation in pension insurance (multiple choice):  ①No pension insurance ②New Rural Pension Insurance ③Commercial Pension Insurance ④Urban Workers' Pension Insurance  ⑤ Urban residents' pension insurance ⑥ Others (please specify) __________ |  |

**II. Evaluation of awareness and satisfaction of contracted family doctor services**

| **serial number** | **Questions and Options** | **responsive** |
| --- | --- | --- |
| 1 | The geographical area and institution of your contracted organization is (e.g. Weifang City, Weicheng District, so-and-so community health service center/township health center): ______________________________ |  |
| 2 | The institution where you sign up for a family doctor is: ① Township Health Center ② Community Health Service Center |  |
| 3 | How much do you know about the family doctor contracting service  ① Very well understood ② Quite well understood ③ Fairly well understood ④ Quite poorly understood ⑤ Very poorly understood |  |
| 4 | How you sign up: ①Voluntary ②Mandatory |  |
| 5 | The members of your contracted family physician team include (check more than one)   1. ①Doctor ②Nurse ③Doctor's assistant ④Pharmacist ⑤Dietician ⑥Health care provider ⑦ Others (please specify) __________ |  |
| 6 | Your current choice of family physician contracted service package is:  ①Basic Service Package ②Primary Package (Maternity, Children, Elderly) ③Intermediate Package ④Advanced Package ⑤Chinese Medicine Package ⑥Unknown ⑦Other (please specify) _________ |  |
| 7 | The fee you pay for the contracted service package is ____￥/year. |  |
| 8 | In the past month, you visited the contracted organization a total of __ times. |  |
| 9 | How long have you been a patient at the contracted facility?   1. ①≤6 months ②6 months-1 year ③1-2 years ④3-4 years ⑤≥5 years |  |
| 10 | Your overall satisfaction with the contracted services provided by the family doctor team:  ① Very satisfied ② Comparatively satisfied ③ General Very satisfied ② Quite satisfied ③ Fair ④ Quite dissatisfied ⑤ Very dissatisfied |  |
| 11 | Are you satisfied with the environment of the contracted organization?  ① Very satisfied ② Comparatively satisfied ③ General Very satisfied ② Quite satisfied ③ Fair ④ Quite dissatisfied ⑤ Very dissatisfied |  |
| 12 | Are you satisfied with the medical ethics of the contracted doctor team?  ① Very satisfied ② Comparatively satisfied ③ General Very satisfied ② Quite satisfied ③ Fair ④ Quite dissatisfied ⑤ Very dissatisfied |  |
| 13 | Are you satisfied with the types of medicines available at the contracted organization?  ① Very satisfied ② Comparatively satisfied ③ General Very satisfied ② Quite satisfied ③ Fair ④ Quite dissatisfied ⑤ Very dissatisfied |  |
| 14 | Are you satisfied with the level of care provided by your contracted doctor?  ① Very satisfied ② Comparatively satisfied ③ General Very satisfied ② Quite satisfied ③ Fair ④ Quite dissatisfied ⑤ Very dissatisfied |  |
| 15 | Are you satisfied with the services of the contracted agency nurses?  ① Very satisfied ② Comparatively satisfied ③ General Very satisfied ② Quite satisfied ③ Fair ④ Quite dissatisfied ⑤ Very dissatisfied |  |
| 16 | Are you satisfied with the medical equipment configuration of the contracted organization?  ① Very satisfied ② Comparatively satisfied ③ General Very satisfied ② Quite satisfied ③ Fair ④ Quite dissatisfied ⑤ Very dissatisfied |  |
| 17 | Are you satisfied with the fees charged by the contracted organization?  ① Very satisfied ② Comparatively satisfied ③ General Very satisfied ② Quite satisfied ③ Fair ④ Quite dissatisfied ⑤ Very dissatisfied |  |

**III. Degree of contact with contracted organizations**

| **serial number** | **Questions and Options** | **responsive** |
| --- | --- | --- |
| 1 | Do you often visit your family doctor when you are not feeling well or have health problems? ①Yes ②No |  |
| 2 | Does your family doctor know about your health condition? ①Yes ②No |  |
| 3 | Do you communicate frequently with your family doctor to keep yourself informed of your health condition? ①Yes ②No |  |

**Ⅳ. Coordination of contracted services - referral services**

| **serial number** | **Questions and Options** | **respondent** |
| --- | --- | --- |
| 1 | Do you get the results of your lab tests?  ①Definitely will ②Probably will ③Probably won't ④Definitely won't ⑤Not sure/Don't know |  |
| 2 | Have you ever visited any other large or specialized hospitals for medical treatment? (If ② or ③, skip to Part V)  ①Yes ②No ③Unsure/can't remember |  |
| 3 | Do you need a referral from a contracted organization before you go to a major or specialty hospital?  ①Yes ②No ③Unsure/Don't know |  |
| 4 | Did your family doctor suggest you go to the specialist or special service?  ①Definitely will ②Probably will ③Probably won't ④Definitely won't ⑤Not sure/Don't know |  |
| 5 | Did your family doctor know you made these visits to the specialist or special service?  ①Definitely will ②Probably will ③Probably won't ④Definitely won't ⑤Not sure/Don't know |  |
| 6 | Did your family doctor discuss with you different places you could have gone to get help with that problem?  ①Definitely will ②Probably will ③Probably won't ④Definitely won't ⑤Not sure/Don't know |  |
| 7 | Did your family doctor help you make the appointment for that visit?  ①Definitely will ②Probably will ③Probably won't ④Definitely won't ⑤Not sure/Don't know |  |
| 8 | Did your family doctor write down any information for the specialist about the reason for the visit?  ①Definitely will ②Probably will ③Probably won't ④Definitely won't ⑤Not sure/Don't know |  |
| 9 | After you went to the specialist or special service, did your family doctor talk with you about what happened at the visit?  ①Definitely will ②Probably will ③Probably won't ④Definitely won't ⑤Not sure/Don't know |  |
| 10 | Did your family doctor seem interested in the quality of care you received from that specialist or specialist service?①Definitely will ②Probably will ③Probably won't ④Definitely won't ⑤Not sure/Don't know |  |
| 11 | When the disease is treated and the condition is stable, whether it will be returned to community institutions for rehabilitation?  ①Definitely will ②Probably will ③Probably won't ④Definitely won't ⑤Not sure/Don't know |  |
| 12 | Whether there are specialists from higher level hospitals in your community?  ①Definitely will ②Probably will ③Probably won't ④Definitely won't ⑤Not sure/Don't know |  |

**Ⅴ. Coordination of contracted services - information systems**

| **serial number** | **Questions and Options** | **responsive** |
| --- | --- | --- |
| 1 | When you go to your family doctor, do you bring any of your own medical records, such as shot records or reports of medical care you had in the past?  ①Definitely will ②Probably will ③Probably won't ④Definitely won't ⑤Not sure/Don't know |  |
| 2 | When you go to your contracted institution, is your medical record always available?  ①Definitely will ②Probably will ③Probably won't ④Definitely won't ⑤Not sure/Don't know |  |
| 3 | Did your family doctor refer to your previous medical records at each visit?  ①Definitely will ②Probably will ③Probably won't ④Definitely won't ⑤Not sure/Don't know |  |
| 4 | Can you see your medical records on the mobile client?  ①Definitely will ②Probably will ③Probably won't ④Definitely won't ⑤Not sure/Don't know |  |
