# Supplementary material for: Study on the evaluation and influencing factors of contracted residents on the coordination of primary medical institutions
Source: Front Public Health. 2024 Jun 4;12:1307765. doi: 10.3389/fpubh.2024.1307765 (PMC11183267; doi:10.3389/fpubh.2024.1307765)
Supplement: Supplementary file 3 [file Table_3.docx]

GET

FILE='C:\Users\Leifxu\Desktop\投稿\潍坊济南菏泽居民库汇总-原库.sav'.

DATASET NAME 数据集1 WINDOW=FRONT.

RELIABILITY

/VARIABLES=G1您能得到签约机构的检查结果吗 G2有没有去过其他大医院看病 G3去大医院前是否需要经过签约机构转诊 G4家庭医生建议您去大医院看病吗 G5家庭医生知道您去过大医院看病吗

G6家庭医生是否与您讨论过其他就医选择 G7转诊时家庭医生会帮您联系专科医院吗 G8转诊时家庭医生会写下您的转诊原因吗 G9您转诊后家庭医生会询问您的治疗情况吗

G10家庭医生对您转诊后的服务质量关注吗 G11病情稳定后是否会转回社区机构康复 G12您社区是否有上级医院的专家来坐诊

/SCALE('ALL VARIABLES') ALL

/MODEL=ALPHA.

| **Case Processing Summary** | | | |
| --- | --- | --- | --- |
|  | | N | % |
| Cases | Valid | 2001 | 51.9 |
|  | Excluded^a^ | 1858 | 48.1 |
|  | Total | 3859 | 100.0 |
| a. Listwise deletion based on all variables in the procedure. | | | |

| **Reliability Statistics** | |
| --- | --- |
| Cronbach's Alpha | N of Items |
| .905 | 12 |

RELIABILITY

/VARIABLES=H1您找家庭医生看病时会携带病历本吗 H2您是否可以在签约机构查看自己的病历资料 H3家庭医生会参考您以前的病历记录吗 H4您是否可在手机客户端看到医疗记录

/SCALE('ALL VARIABLES') ALL

/MODEL=ALPHA.

Reliability

Scale: ALL VARIABLES

| **Case Processing Summary** | | | |
| --- | --- | --- | --- |
|  | | N | % |
| Cases | Valid | 3859 | 100.0 |
|  | Excluded^a^ | 0 | .0 |
|  | Total | 3859 | 100.0 |
| a. Listwise deletion based on all variables in the procedure. | | | |

| **Reliability Statistics** | |
| --- | --- |
| Cronbach's Alpha | N of Items |
| .790 | 4 |

FACTOR

/VARIABLES G1您能得到签约机构的检查结果吗 G2有没有去过其他大医院看病 G3去大医院前是否需要经过签约机构转诊 G4家庭医生建议您去大医院看病吗 G5家庭医生知道您去过大医院看病吗

G6家庭医生是否与您讨论过其他就医选择 G7转诊时家庭医生会帮您联系专科医院吗 G8转诊时家庭医生会写下您的转诊原因吗 G9您转诊后家庭医生会询问您的治疗情况吗

G10家庭医生对您转诊后的服务质量关注吗 G11病情稳定后是否会转回社区机构康复 G12您社区是否有上级医院的专家来坐诊

/MISSING LISTWISE

/ANALYSIS G1您能得到签约机构的检查结果吗 G2有没有去过其他大医院看病 G3去大医院前是否需要经过签约机构转诊 G4家庭医生建议您去大医院看病吗 G5家庭医生知道您去过大医院看病吗

G6家庭医生是否与您讨论过其他就医选择 G7转诊时家庭医生会帮您联系专科医院吗 G8转诊时家庭医生会写下您的转诊原因吗 G9您转诊后家庭医生会询问您的治疗情况吗

G10家庭医生对您转诊后的服务质量关注吗 G11病情稳定后是否会转回社区机构康复 G12您社区是否有上级医院的专家来坐诊

/PRINT INITIAL KMO EXTRACTION ROTATION

/FORMAT SORT BLANK(.5)

/PLOT EIGEN

/CRITERIA MINEIGEN(1) ITERATE(25)

/EXTRACTION PC

/CRITERIA ITERATE(25)

/ROTATION VARIMAX

/METHOD=CORRELATION.

- - - - - - - - - - - - - - - - - - - - - - - - F A C T O R A N A L Y S I S - - - - - - - - - - - - - - - - - - - - - - - -

| **KMO and Bartlett's Test** | | |
| --- | --- | --- |
| Kaiser-Meyer-Olkin Measure of Sampling Adequacy. | | .926 |
| Bartlett's Test of Sphericity | Approx. Chi-Square | 12832.252 |
|  | df | 66 |
|  | Sig. | .000 |

FACTOR

/VARIABLES H1您找家庭医生看病时会携带病历本吗 H2您是否可以在签约机构查看自己的病历资料 H3家庭医生会参考您以前的病历记录吗 H4您是否可在手机客户端看到医疗记录

/MISSING LISTWISE

/ANALYSIS H1您找家庭医生看病时会携带病历本吗 H2您是否可以在签约机构查看自己的病历资料 H3家庭医生会参考您以前的病历记录吗 H4您是否可在手机客户端看到医疗记录

/PRINT INITIAL KMO EXTRACTION ROTATION

/FORMAT SORT BLANK(.5)

/PLOT EIGEN

/CRITERIA MINEIGEN(1) ITERATE(25)

/EXTRACTION PC

/CRITERIA ITERATE(25)

/ROTATION VARIMAX

/METHOD=CORRELATION.

| **KMO and Bartlett's Test** | | |
| --- | --- | --- |
| Kaiser-Meyer-Olkin Measure of Sampling Adequacy. | | .781 |
| Bartlett's Test of Sphericity | Approx. Chi-Square | 5394.140 |
|  | df | 6 |
|  | Sig. | .000 |

GET

FILE='C:\Users\Leifxu\Desktop\投稿\潍坊济南菏泽居民库汇总-原库.sav'.

DATASET NAME 数据集1 WINDOW=FRONT.

RECODE G1您能得到签约机构的检查结果吗 (1=4) (2=3) (3=2) (4=1) (5=2) INTO G1.

EXECUTE.

RECODE G4家庭医生建议您去大医院看病吗 (1=4) (2=3) (3=2) (4=1) (5=2) (MISSING=0) INTO G4.

EXECUTE.

RECODE G5家庭医生知道您去过大医院看病吗 (1=4) (2=3) (3=2) (4=1) (5=2) (MISSING=0) INTO G5.

EXECUTE.

RECODE G6家庭医生是否与您讨论过其他就医选择 (1=4) (2=3) (3=2) (4=1) (5=2) (MISSING=0) INTO G6.

EXECUTE.

RECODE G7转诊时家庭医生会帮您联系专科医院吗 (1=4) (2=3) (3=2) (4=1) (5=2) (MISSING=0) INTO G7.

EXECUTE.

RECODE G8转诊时家庭医生会写下您的转诊原因吗 (1=4) (2=3) (3=2) (4=1) (5=2) (MISSING=0) INTO G8.

EXECUTE.

RECODE G9您转诊后家庭医生会询问您的治疗情况吗 (1=4) (2=3) (3=2) (4=1) (5=2) (MISSING=0) INTO G9.

EXECUTE.

RECODE G10家庭医生对您转诊后的服务质量关注吗 (1=4) (2=3) (3=2) (4=1) (5=2) (MISSING=0) INTO G10.

EXECUTE.

RECODE G11病情稳定后是否会转回社区机构康复 (1=4) (2=3) (3=2) (4=1) (5=2) (MISSING=0) INTO G11.

EXECUTE.

RECODE G12您社区是否有上级医院的专家来坐诊 (1=4) (2=3) (3=2) (4=1) (5=2) (MISSING=0) INTO G12.

EXECUTE.

RECODE H1您找家庭医生看病时会携带病历本吗 (1=4) (2=3) (3=2) (4=1) (5=2) (MISSING=0) INTO h1.

EXECUTE.

RECODE H2您是否可以在签约机构查看自己的病历资料 (1=4) (2=3) (3=2) (4=1) (5=2) (MISSING=0) INTO H2.

EXECUTE.

RECODE H3家庭医生会参考您以前的病历记录吗 (1=4) (2=3) (3=2) (4=1) (5=2) (MISSING=0) INTO H3.

EXECUTE.

RECODE H4您是否可在手机客户端看到医疗记录 (1=4) (2=3) (3=2) (4=1) (5=2) (MISSING=0) INTO H4.

EXECUTE.

COMPUTE 信息=(h1 + H2 + H3 + H4) / 4.

EXECUTE.

DESCRIPTIVES VARIABLES=信息

/STATISTICS=MEAN STDDEV MIN MAX.

| **Descriptive Statistics** | | | | | |
| --- | --- | --- | --- | --- | --- |
|  | N | Minimum | Maximum | Mean | Std. Deviation |
| M1coordinationrefferal | 3859 | 1.00 | 4.00 | 3.6047 | .57744 |
| M2coordinationinformation | 3859 | 1.00 | 4.00 | 3.3377 | .64926 |
| M3Coordinationscore | 3859 | 1.00 | 4.00 | 3.4112 | .57139 |
| Valid N (listwise) | 3859 |  |  |  |  |

DESCRIPTIVES VARIABLES=G1 G4 G5 G6 G7 G8 G9 G10 G11 G12 h1 H2 H3 H4

/STATISTICS=MEAN STDDEV MIN MAX.

| **Descriptive Statistics** | | | | | |
| --- | --- | --- | --- | --- | --- |
|  | N | Minimum | Maximum | Mean | Std. Deviation |
| G1 | 3859 | 1.00 | 4.00 | 3.7476 | .54506 |
| G4 | 2001 | 1.00 | 4.00 | 3.5032 | .64816 |
| G5 | 2001 | 1.00 | 4.00 | 3.5342 | .72798 |
| G6 | 2001 | 1.00 | 4.00 | 3.4543 | .74164 |
| G7 | 2001 | 1.00 | 4.00 | 3.4613 | .76657 |
| G8 | 2004 | 1.00 | 4.00 | 3.5379 | .72682 |
| G9 | 2004 | 1.00 | 4.00 | 3.5474 | .72626 |
| G10 | 2004 | 1.00 | 4.00 | 3.4416 | .79632 |
| G11 | 2004 | 1.00 | 4.00 | 3.5509 | .67173 |
| G12 | 2004 | 1.00 | 4.00 | 3.3473 | .84856 |
| h1 | 3859 | 1.00 | 4.00 | 3.4325 | .85240 |
| H2 | 3859 | 1.00 | 4.00 | 3.4294 | .73980 |
| H3 | 3859 | 1.00 | 4.00 | 3.5670 | .64307 |
| H4 | 3859 | 1.00 | 4.00 | 2.9217 | 1.03395 |
| Valid N (listwise) | 2001 |  |  |  |  |

SORT CASES BY G7 (A).

SORT CASES BY G7 (D).

RECODE A2年龄 (Lowest thru 18=1) (19 thru 29=2) (30 thru 39=3) (40 thru 49=4) (50 thru 59=5) (60 thru

Highest=6) INTO Agegroup.

EXECUTE.

DESCRIPTIVES VARIABLES=Agegroup

/STATISTICS=MEAN STDDEV MIN MAX.

FREQUENCIES VARIABLES=Agegroup

/ORDER=ANALYSIS.

| **Agegroup** | | | | | |
| --- | --- | --- | --- | --- | --- |
|  | | Frequency | Percent | Valid Percent | Cumulative Percent |
| Valid | 1.00 | 65 | 1.7 | 1.7 | 1.7 |
|  | 2.00 | 333 | 8.6 | 8.6 | 10.3 |
|  | 3.00 | 507 | 13.1 | 13.1 | 23.5 |
|  | 4.00 | 900 | 23.3 | 23.3 | 46.8 |
|  | 5.00 | 810 | 21.0 | 21.0 | 67.8 |
|  | 6.00 | 1244 | 32.2 | 32.2 | 100.0 |
|  | Total | 3859 | 100.0 | 100.0 |  |

RECODE A2年龄 (30 thru 39=3) (40 thru 49=4) (50 thru 59=5) (60 thru Highest=6) (Lowest thru 17=1) (18

thru 29=2) INTO Agegroup.

EXECUTE.

FREQUENCIES VARIABLES=Agegroup

/ORDER=ANALYSIS.

RECODE A6个人收入 (Lowest thru 1999=1) (2000 thru 4999=2) (5000 thru Highest=3) INTO A6personnalincome.

EXECUTE.

RECODE A5职业 (9=1) (7=2) (8=3) (Lowest thru 6=1) INTO A5Occupationgroup.

EXECUTE.

RECODE 地市 (1=2) (2=1) (3=3) INTO localeconomylevel.

EXECUTE.

ONEWAY M3Coordinationscore BY localeconomylevel

/MISSING ANALYSIS.

| **Descriptives** | | | | | | | | |
| --- | --- | --- | --- | --- | --- | --- | --- | --- |
| M3Coordinationscore | | | | | | | | |
|  | N | Mean | Std. Deviation | Std. Error | 95% Confidence Interval for Mean | | Minimum | Maximum |
|  |  |  |  |  | Lower Bound | Upper Bound |  |  |
| 1.00 | 1333 | 3.5857 | .53948 | .01478 | 3.5567 | 3.6147 | 1.00 | 4.00 |
| 2.00 | 1428 | 3.6139 | .42696 | .01130 | 3.5917 | 3.6360 | 1.63 | 4.00 |
| 3.00 | 1098 | 3.1465 | .54563 | .01647 | 3.1142 | 3.1788 | 1.53 | 4.00 |
| Total | 3859 | 3.4712 | .54280 | .00874 | 3.4540 | 3.4883 | 1.00 | 4.00 |

ONEWAY M3Coordinationscore BY localeconomylevel

/STATISTICS DESCRIPTIVES HOMOGENEITY

/PLOT MEANS

/MISSING ANALYSIS

/POSTHOC=LSD T2 ALPHA(0.05).

ONEWAY M3Coordinationscore BY localeconomylevel

/STATISTICS DESCRIPTIVES HOMOGENEITY

/PLOT MEANS

/MISSING ANALYSIS

/POSTHOC=LSD T2 ALPHA(0.05).

ONEWAY M3Coordinationscore BY Agegroup

/STATISTICS DESCRIPTIVES HOMOGENEITY

/PLOT MEANS

/MISSING ANALYSIS

/POSTHOC=LSD T2 ALPHA(0.05).

| **Descriptives** | | | | | | | | |
| --- | --- | --- | --- | --- | --- | --- | --- | --- |
| M3Coordinationscore | | | | | | | | |
|  | N | Mean | Std. Deviation | Std. Error | 95% Confidence Interval for Mean | | Minimum | Maximum |
|  |  |  |  |  | Lower Bound | Upper Bound |  |  |
| 1.00 | 39 | 3.2982 | .72293 | .11576 | 3.0638 | 3.5325 | 1.80 | 4.00 |
| 2.00 | 359 | 3.1736 | .73552 | .03882 | 3.0973 | 3.2500 | 1.20 | 4.00 |
| 3.00 | 507 | 3.5638 | .50969 | .02264 | 3.5193 | 3.6083 | 1.79 | 4.00 |
| 4.00 | 900 | 3.4831 | .51449 | .01715 | 3.4494 | 3.5168 | 1.80 | 4.00 |
| 5.00 | 810 | 3.4744 | .51206 | .01799 | 3.4390 | 3.5097 | 1.71 | 4.00 |
| 6.00 | 1244 | 3.3279 | .57810 | .01639 | 3.2958 | 3.3601 | 1.00 | 4.00 |
| Total | 3859 | 3.4112 | .57139 | .00920 | 3.3932 | 3.4292 | 1.00 | 4.00 |

| **ANOVA** | | | | | |
| --- | --- | --- | --- | --- | --- |
| M3Coordinationscore | | | | | |
|  | Sum of Squares | df | Mean Square | F | Sig. |
| Between Groups | 49.078 | 5 | 9.816 | 31.244 | .000 |
| Within Groups | 1210.487 | 3853 | .314 |  |  |
| Total | 1259.565 | 3858 |  |  |  |

ONEWAY M3Coordinationscore BY A4婚姻状况

/STATISTICS DESCRIPTIVES HOMOGENEITY

/PLOT MEANS

/MISSING ANALYSIS

/POSTHOC=LSD T2 ALPHA(0.05).

| **Descriptives** | | | | | | | | |
| --- | --- | --- | --- | --- | --- | --- | --- | --- |
| M3Coordinationscore | | | | | | | | |
|  | N | Mean | Std. Deviation | Std. Error | 95% Confidence Interval for Mean | | Minimum | Maximum |
|  |  |  |  |  | Lower Bound | Upper Bound |  |  |
| 未婚 | 346 | 3.1038 | .73667 | .03960 | 3.0259 | 3.1817 | 1.20 | 4.00 |
| 已婚 | 3257 | 3.4571 | .53713 | .00941 | 3.4386 | 3.4755 | 1.00 | 4.00 |
| 离婚 | 36 | 3.2409 | .61604 | .10267 | 3.0324 | 3.4493 | 1.80 | 4.00 |
| 丧偶 | 220 | 3.2435 | .57700 | .03890 | 3.1668 | 3.3202 | 1.40 | 4.00 |
| Total | 3859 | 3.4112 | .57139 | .00920 | 3.3932 | 3.4292 | 1.00 | 4.00 |

| **ANOVA** | | | | | |
| --- | --- | --- | --- | --- | --- |
| M3Coordinationscore | | | | | |
|  | Sum of Squares | df | Mean Square | F | Sig. |
| Between Groups | 46.775 | 3 | 15.592 | 49.560 | .000 |
| Within Groups | 1212.790 | 3855 | .315 |  |  |
| Total | 1259.565 | 3858 |  |  |  |

ONEWAY M3Coordinationscore BY A3文化程度

/STATISTICS DESCRIPTIVES HOMOGENEITY

/PLOT MEANS

/MISSING ANALYSIS

/POSTHOC=LSD T2 ALPHA(0.05).

| **Descriptives** | | | | | | | | |
| --- | --- | --- | --- | --- | --- | --- | --- | --- |
| M3Coordinationscore | | | | | | | | |
|  | N | Mean | Std. Deviation | Std. Error | 95% Confidence Interval for Mean | | Minimum | Maximum |
|  |  |  |  |  | Lower Bound | Upper Bound |  |  |
| 小学及以下 | 1204 | 3.2541 | .57891 | .01668 | 3.2214 | 3.2869 | 1.00 | 4.00 |
| 初中 | 1276 | 3.5291 | .50671 | .01419 | 3.5013 | 3.5570 | 1.80 | 4.00 |
| 高中（中专） | 657 | 3.5019 | .50742 | .01980 | 3.4630 | 3.5408 | 1.80 | 4.00 |
| 大专 | 358 | 3.5127 | .56557 | .02989 | 3.4539 | 3.5715 | 1.20 | 4.00 |
| 本科及以上 | 364 | 3.2537 | .70488 | .03695 | 3.1810 | 3.3263 | 1.60 | 4.00 |
| Total | 3859 | 3.4112 | .57139 | .00920 | 3.3932 | 3.4292 | 1.00 | 4.00 |

| **ANOVA** | | | | | |
| --- | --- | --- | --- | --- | --- |
| M3Coordinationscore | | | | | |
|  | Sum of Squares | df | Mean Square | F | Sig. |
| Between Groups | 65.583 | 4 | 16.396 | 52.923 | .000 |
| Within Groups | 1193.982 | 3854 | .310 |  |  |
| Total | 1259.565 | 3858 |  |  |  |

ONEWAY M3Coordinationscore BY A5Occupationgroup

/STATISTICS DESCRIPTIVES HOMOGENEITY

/PLOT MEANS

/MISSING ANALYSIS

/POSTHOC=LSD T2 ALPHA(0.05).

| **Descriptives** | | | | | | | | |
| --- | --- | --- | --- | --- | --- | --- | --- | --- |
| M3Coordinationscore | | | | | | | | |
|  | N | Mean | Std. Deviation | Std. Error | 95% Confidence Interval for Mean | | Minimum | Maximum |
|  |  |  |  |  | Lower Bound | Upper Bound |  |  |
| 1.00 | 3393 | 3.4359 | .54748 | .00940 | 3.4175 | 3.4544 | 1.40 | 4.00 |
| 2.00 | 214 | 2.9808 | .74564 | .05097 | 2.8804 | 3.0813 | 1.20 | 4.00 |
| 3.00 | 252 | 3.4435 | .57317 | .03611 | 3.3724 | 3.5146 | 1.00 | 4.00 |
| Total | 3859 | 3.4112 | .57139 | .00920 | 3.3932 | 3.4292 | 1.00 | 4.00 |

| **ANOVA** | | | | | |
| --- | --- | --- | --- | --- | --- |
| M3Coordinationscore | | | | | |
|  | Sum of Squares | df | Mean Square | F | Sig. |
| Between Groups | 41.973 | 2 | 20.987 | 66.463 | .000 |
| Within Groups | 1217.592 | 3856 | .316 |  |  |
| Total | 1259.565 | 3858 |  |  |  |

ONEWAY M3Coordinationscore BY A6personnalincome

/STATISTICS DESCRIPTIVES HOMOGENEITY

/PLOT MEANS

/MISSING ANALYSIS

/POSTHOC=LSD T2 ALPHA(0.05).

| **Descriptives** | | | | | | | | |
| --- | --- | --- | --- | --- | --- | --- | --- | --- |
| M3Coordinationscore | | | | | | | | |
|  | N | Mean | Std. Deviation | Std. Error | 95% Confidence Interval for Mean | | Minimum | Maximum |
|  |  |  |  |  | Lower Bound | Upper Bound |  |  |
| 1.00 | 1765 | 3.3124 | .60656 | .01444 | 3.2841 | 3.3407 | 1.20 | 4.00 |
| 2.00 | 1549 | 3.5106 | .51353 | .01305 | 3.4850 | 3.5362 | 1.00 | 4.00 |
| 3.00 | 545 | 3.4485 | .55743 | .02388 | 3.4016 | 3.4954 | 1.79 | 4.00 |
| Total | 3859 | 3.4112 | .57139 | .00920 | 3.3932 | 3.4292 | 1.00 | 4.00 |

| **ANOVA** | | | | | |
| --- | --- | --- | --- | --- | --- |
| M3Coordinationscore | | | | | |
|  | Sum of Squares | df | Mean Square | F | Sig. |
| Between Groups | 33.315 | 2 | 16.658 | 52.381 | .000 |
| Within Groups | 1226.250 | 3856 | .318 |  |  |
| Total | 1259.565 | 3858 |  |  |  |

ONEWAY M3Coordinationscore BY A7您身体健康状况

/STATISTICS DESCRIPTIVES HOMOGENEITY

/PLOT MEANS

/MISSING ANALYSIS

/POSTHOC=LSD T2 ALPHA(0.05).

| **Descriptives** | | | | | | | | |
| --- | --- | --- | --- | --- | --- | --- | --- | --- |
| M3Coordinationscore | | | | | | | | |
|  | N | Mean | Std. Deviation | Std. Error | 95% Confidence Interval for Mean | | Minimum | Maximum |
|  |  |  |  |  | Lower Bound | Upper Bound |  |  |
| 非常好 | 1028 | 3.5541 | .52998 | .01653 | 3.5216 | 3.5865 | 1.20 | 4.00 |
| 比较好 | 1631 | 3.3956 | .55854 | .01383 | 3.3684 | 3.4227 | 1.60 | 4.00 |
| 一般 | 957 | 3.3491 | .59236 | .01915 | 3.3115 | 3.3867 | 1.40 | 4.00 |
| 比较差 | 228 | 3.1705 | .57983 | .03840 | 3.0948 | 3.2462 | 1.80 | 4.00 |
| 非常差 | 15 | 2.9371 | .81014 | .20918 | 2.4885 | 3.3858 | 1.00 | 4.00 |
| Total | 3859 | 3.4112 | .57139 | .00920 | 3.3932 | 3.4292 | 1.00 | 4.00 |

| **ANOVA** | | | | | |
| --- | --- | --- | --- | --- | --- |
| M3Coordinationscore | | | | | |
|  | Sum of Squares | df | Mean Square | F | Sig. |
| Between Groups | 41.647 | 4 | 10.412 | 32.947 | .000 |
| Within Groups | 1217.918 | 3854 | .316 |  |  |
| Total | 1259.565 | 3858 |  |  |  |

T-TEST GROUPS=B2您的签约机构是(1 2)

/MISSING=ANALYSIS

/VARIABLES=M3Coordinationscore

/CRITERIA=CI(.95).

| **Group Statistics** | | | | | |
| --- | --- | --- | --- | --- | --- |
|  | B2您的签约机构是 | N | Mean | Std. Deviation | Std. Error Mean |
| M3Coordinationscore | 乡镇卫生院 | 2086 | 3.4825 | .51104 | .01119 |
|  | 社区卫生服务中心 | 1773 | 3.3273 | .62491 | .01484 |

| **Independent Samples Test** | | | | | | | | | | |
| --- | --- | --- | --- | --- | --- | --- | --- | --- | --- | --- |
|  | | Levene's Test for Equality of Variances | | t-test for Equality of Means | | | | | | |
|  |  | F | Sig. | t | df | Sig. (2-tailed) | Mean Difference | Std. Error Difference | 95% Confidence Interval of the Difference | |
|  |  |  |  |  |  |  |  |  | Lower | Upper |
| M3Coordinationscore | Equal variances assumed | 115.721 | .000 | 8.481 | 3857 | .000 | .15511 | .01829 | .11925 | .19097 |
|  | Equal variances not assumed |  |  | 8.345 | 3419.921 | .000 | .15511 | .01859 | .11867 | .19155 |

T-TEST GROUPS=A1性别(1 2)

/MISSING=ANALYSIS

/VARIABLES=M3Coordinationscore

/CRITERIA=CI(.95).

| **Group Statistics** | | | | | |
| --- | --- | --- | --- | --- | --- |
|  | A1性别 | N | Mean | Std. Deviation | Std. Error Mean |
| M3Coordinationscore | 男 | 1802 | 3.4575 | .56022 | .01320 |
|  | 女 | 2057 | 3.3707 | .57809 | .01275 |

| **Independent Samples Test** | | | | | | | | | | |
| --- | --- | --- | --- | --- | --- | --- | --- | --- | --- | --- |
|  | | Levene's Test for Equality of Variances | | t-test for Equality of Means | | | | | | |
|  |  | F | Sig. | t | df | Sig. (2-tailed) | Mean Difference | Std. Error Difference | 95% Confidence Interval of the Difference | |
|  |  |  |  |  |  |  |  |  | Lower | Upper |
| M3Coordinationscore | Equal variances assumed | 2.942 | .086 | 4.720 | 3857 | .000 | .08679 | .01839 | .05074 | .12283 |
|  | Equal variances not assumed |  |  | 4.730 | 3817.992 | .000 | .08679 | .01835 | .05082 | .12276 |

T-TEST GROUPS=A8您是否有已经确诊过的慢性病(1 2)

/MISSING=ANALYSIS

/VARIABLES=M3Coordinationscore

/CRITERIA=CI(.95).

| **Group Statistics** | | | | | |
| --- | --- | --- | --- | --- | --- |
|  | A8您是否有已经确诊过的慢性病 | N | Mean | Std. Deviation | Std. Error Mean |
| M3Coordinationscore | 是 | 1370 | 3.3671 | .57306 | .01548 |
|  | 否 | 2489 | 3.4355 | .56912 | .01141 |

| **Independent Samples Test** | | | | | | | | | | |
| --- | --- | --- | --- | --- | --- | --- | --- | --- | --- | --- |
|  | | Levene's Test for Equality of Variances | | t-test for Equality of Means | | | | | | |
|  |  | F | Sig. | t | df | Sig. (2-tailed) | Mean Difference | Std. Error Difference | 95% Confidence Interval of the Difference | |
|  |  |  |  |  |  |  |  |  | Lower | Upper |
| M3Coordinationscore | Equal variances assumed | 2.128 | .145 | -3.562 | 3857 | .000 | -.06837 | .01919 | -.10600 | -.03075 |
|  | Equal variances not assumed |  |  | -3.555 | 2804.116 | .000 | -.06837 | .01923 | -.10608 | -.03067 |

T-TEST GROUPS=A10.1没有参加医疗保险(1 2)

/MISSING=ANALYSIS

/VARIABLES=M3Coordinationscore

/CRITERIA=CI(.95).

| **Group Statistics** | | | | | |
| --- | --- | --- | --- | --- | --- |
|  | A10.1没有参加医疗保险 | N | Mean | Std. Deviation | Std. Error Mean |
| M3Coordinationscore | 是 | 74 | 3.1450 | .71558 | .08318 |
|  | 否 | 3785 | 3.4164 | .56708 | .00922 |

| **Independent Samples Test** | | | | | | | | | | |
| --- | --- | --- | --- | --- | --- | --- | --- | --- | --- | --- |
|  | | Levene's Test for Equality of Variances | | t-test for Equality of Means | | | | | | |
|  |  | F | Sig. | t | df | Sig. (2-tailed) | Mean Difference | Std. Error Difference | 95% Confidence Interval of the Difference | |
|  |  |  |  |  |  |  |  |  | Lower | Upper |
| M3Coordinationscore | Equal variances assumed | 14.183 | .000 | -4.055 | 3857 | .000 | -.27141 | .06693 | -.40265 | -.14018 |
|  | Equal variances not assumed |  |  | -3.243 | 74.803 | .002 | -.27141 | .08369 | -.43815 | -.10468 |

T-TEST GROUPS=A11.1没有参加养老保险(1 2)

/MISSING=ANALYSIS

/VARIABLES=M3Coordinationscore

/CRITERIA=CI(.95).

| **Group Statistics** | | | | | |
| --- | --- | --- | --- | --- | --- |
|  | A11.1没有参加养老保险 | N | Mean | Std. Deviation | Std. Error Mean |
| M3Coordinationscore | 是 | 586 | 3.2358 | .65107 | .02690 |
|  | 否 | 3272 | 3.4425 | .55020 | .00962 |

| **Independent Samples Test** | | | | | | | | | | |
| --- | --- | --- | --- | --- | --- | --- | --- | --- | --- | --- |
|  | | Levene's Test for Equality of Variances | | t-test for Equality of Means | | | | | | |
|  |  | F | Sig. | t | df | Sig. (2-tailed) | Mean Difference | Std. Error Difference | 95% Confidence Interval of the Difference | |
|  |  |  |  |  |  |  |  |  | Lower | Upper |
| M3Coordinationscore | Equal variances assumed | 43.201 | .000 | -8.132 | 3856 | .000 | -.20670 | .02542 | -.25653 | -.15686 |
|  | Equal variances not assumed |  |  | -7.236 | 742.042 | .000 | -.20670 | .02856 | -.26277 | -.15062 |

RECODE localeconomylevel (2=1) (ELSE=0) INTO general.

EXECUTE.

RECODE localeconomylevel (2=1) (ELSE=0) INTO general.

EXECUTE.

RECODE localeconomylevel (3=1) (ELSE=0) INTO poor.

EXECUTE.

RECODE B2您的签约机构是 (1=1) (ELSE=0) INTO townshiphealthcenter.

EXECUTE.

RECODE A3文化程度 (2=1) (ELSE=0) INTO juniorhighschool.

EXECUTE.

RECODE A3文化程度 (3=1) (ELSE=0) INTO highschool.

EXECUTE.

RECODE A3文化程度 (4=1) (ELSE=0) INTO juniorcollege.

EXECUTE.

RECODE A3文化程度 (5=1) (ELSE=0) INTO bachelorabove.

EXECUTE.

RECODE A4婚姻状况 (2=1) (ELSE=0) INTO married.

EXECUTE.

RECODE A4婚姻状况 (3=1) (ELSE=0) INTO divorce.

EXECUTE.

RECODE A4婚姻状况 (4=1) (ELSE=0) INTO widowed.

EXECUTE.

RECODE A5Occupationgroup (2=1) (ELSE=0) INTO schoolstudents.

EXECUTE.

RECODE A5Occupationgroup (3=1) (ELSE=0) INTO retired.

EXECUTE.

RECODE A7您身体健康状况 (2=1) (ELSE=0) INTO good.

EXECUTE.

RECODE A7您身体健康状况 (3=1) (ELSE=0) INTO generalhealthy.

EXECUTE.

RECODE A7您身体健康状况 (4=1) (ELSE=0) INTO poorhealthy.

EXECUTE.

RECODE A7您身体健康状况 (5=1) (ELSE=0) INTO verypoor.

EXECUTE.

RECODE A8您是否有已经确诊过的慢性病 (1=1) (ELSE=0) INTO chronicdiseasediagnosed.

EXECUTE.

RECODE A10.1没有参加医疗保险 (1=1) (ELSE=0) INTO healthinsurance.

EXECUTE.

RECODE A11.1没有参加养老保险 (1=1) (ELSE=0) INTO endowmentinsurance.

EXECUTE.

RECODE A1性别 (1=1) (ELSE=0) INTO male.

EXECUTE.

REGRESSION

/MISSING LISTWISE

/STATISTICS COEFF OUTS CI(95) R ANOVA COLLIN TOL

/CRITERIA=PIN(.05) POUT(.10)

/NOORIGIN

/DEPENDENT M3Coordinationscore

/METHOD=ENTER general poor male townshiphealthcenter juniorhighschool highschool juniorcollege

bachelorabove married divorce widowed A6personnalincome schoolstudents retired good generalhealthy

poorhealthy verypoor chronicdiseasediagnosed healthinsurance endowmentinsurance.

RECODE localeconomylevel (2=1) (ELSE=0) INTO general.

EXECUTE.

RECODE localeconomylevel (3=1) (ELSE=0) INTO poor.

EXECUTE.

REGRESSION

/MISSING LISTWISE

/STATISTICS COEFF OUTS CI(95) R ANOVA

/CRITERIA=PIN(.05) POUT(.10)

/NOORIGIN

/DEPENDENT M3Coordinationscore

/METHOD=ENTER general poor townshiphealthcenter male A2年龄 juniorhighschool highschool

juniorcollege bachelorabove married divorce widowed A6个人收入 schoolstudents retired good

generalhealthy poorhealthy verypoor chronicdiseasediagnosed healthinsurance endowmentinsurance.

| Coefficientsa | | | | | | | | |
| --- | --- | --- | --- | --- | --- | --- | --- | --- |
| Model | | Unstandardized Coefficients | | Standardized Coefficients | t | Sig. | 95.0% Confidence Interval for B | |
|  |  | B | Std. Error | Beta |  |  | Lower Bound | Upper Bound |
| 1 | (Constant) | 3.489 | .065 |  | 53.899 | .000 | 3.362 | 3.616 |
|  | general | -.003 | .019 | -.003 | -.173 | .863 | -.041 | .035 |
|  | poor | -.479 | .022 | -.378 | -21.685 | .000 | -.522 | -.436 |
|  | townshiphealthcenter | .086 | .018 | .075 | 4.875 | .000 | .051 | .120 |
|  | male | .040 | .017 | .035 | 2.378 | .017 | .007 | .072 |
|  | A2年龄 | -.001 | .001 | -.019 | -.787 | .431 | -.002 | .001 |
|  | juniorhighschool | .076 | .024 | .062 | 3.199 | .001 | .029 | .122 |
|  | highschool | .025 | .029 | .017 | .858 | .391 | -.032 | .083 |
|  | juniorcollege | .108 | .037 | .055 | 2.933 | .003 | .036 | .181 |
|  | bachelorabove | .017 | .041 | .009 | .415 | .679 | -.063 | .097 |
|  | married | .166 | .046 | .105 | 3.585 | .000 | .075 | .257 |
|  | divorce | -.065 | .094 | -.011 | -.686 | .493 | -.250 | .120 |
|  | widowed | .166 | .060 | .068 | 2.778 | .006 | .049 | .284 |
|  | A6个人收入 | -7.908E-6 | .000 | -.059 | -3.913 | .000 | .000 | .000 |
|  | schoolstudents | -.183 | .058 | -.073 | -3.167 | .002 | -.296 | -.070 |
|  | retired | .000 | .035 | .000 | .012 | .991 | -.069 | .069 |
|  | good | -.167 | .021 | -.144 | -8.098 | .000 | -.208 | -.127 |
|  | generalhealthy | -.196 | .025 | -.148 | -7.858 | .000 | -.245 | -.147 |
|  | poorhealthy | -.250 | .040 | -.103 | -6.196 | .000 | -.329 | -.171 |
|  | verypoor | -.418 | .131 | -.045 | -3.192 | .001 | -.674 | -.161 |
|  | chronicdiseasediagnosed | .039 | .020 | .033 | 1.922 | .055 | -.001 | .079 |
|  | healthinsurance | -.068 | .061 | -.016 | -1.125 | .261 | -.187 | .051 |
|  | endowmentinsurance | -.051 | .026 | -.032 | -1.992 | .046 | -.101 | -.001 |
| a. Dependent Variable: M3Coordinationscore | | | | | | | | |
